# Michael J. Gibney (1948–2024): a man of his time who had the time of his life

**DOI:** 10.1017/S136898002400185X

**Published:** 2025-01-23

**Authors:** Christine M. Williams

**Affiliations:** The Hugh Sinclair Unit of Human Nutrition, University of Reading, Reading, UK

**Figure:**
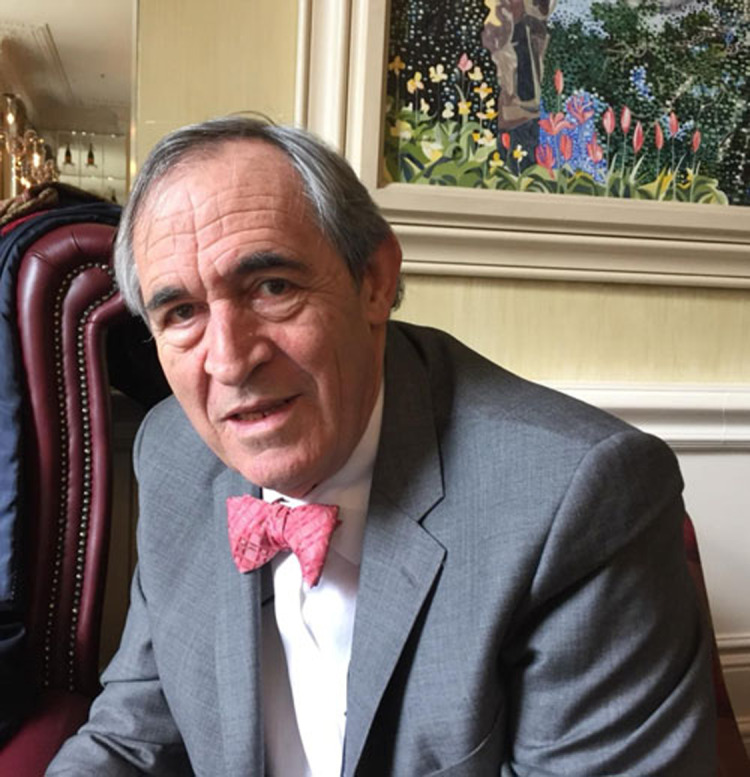

Professor Mike Gibney, who died on 23 February 2024, was born in 1948 into a family of six children living on the north side of Dublin, to a father trained as a carpenter, who became a trade union leader, and a mother, formerly a nurse, who, like many mothers of that era, was highly ambitious for her children. That ambition proved to be very well-founded as Ireland would soon be entering a period of rapid economic, social and cultural change. As well as the post-war developments that were benefiting many developed countries of Western Europe, the loosening of ties with the Catholic Church and the yet-to-be-realised impact of joining the European Union in 1973 would prove to be transformative for the Irish population. Mike Gibney, a child of that time, would progress to become one of the most outstanding academics and science communicators of his generation. For many of his colleagues, students and collaborators, his gift for friendship, love of conversation and conviviality and the importance he gave to mentoring and developing other people will live long in their memories and in their lives.

Today, it might seem inevitable that a bright, articulate and focused young man was more than likely to succeed in whatever career he chose. But Mike’s story of how he came to follow an academic career in human nutrition, from an undergraduate degree in agricultural chemistry to founding director of the Institute of Food and Health at University College Dublin, did not follow a linear process. Nevertheless, in his autobiography, ‘A Life in Food: A Grain of Salt and Some Humble Pie’^(1)^, he lays down some pointers, which suggest his subsequent career was at least in part determined by his undergraduate preference to study animal rather than plant biology, and a master’s project involving the analysis of the fatty acid composition of adipose tissue of growing lambs, indicators of interests that foreshadowed a significant part of his research in later years.

Mike was offered several PhD posts and, with his new wife Jo, chose to leave Dublin in 1973 to study for his PhD in Sydney, Australia under Dr Don Walker, a leading researcher in the nutrition of neonatal lambs. His experimental research was technically demanding, but the outcomes were successful^(2)^, and Mike made relative light of his heavy workload despite the additional responsibilities he held as a teaching fellow and later an assistant lecturer. Mike and Jo left Sydney in 1976 with Mike’s PhD in hand and two children (plus one on the way). This was a formative and productive time, with Mike learning the tools of his trade as well as founding a family that would prove to be the core of his life for the next 50 years.

His next appointment back in Dublin was less auspicious but ultimately determined his migration towards human nutrition research. His post was funded by a government post-doctoral fellowship for returning Irish PhD students who had studied abroad. So limited were the facilities at the Agricultural Institute which hosted him and the supervision non-existent that Mike was left pretty much to his own devices and in his own words ‘began to dabble in human nutrition’. Mike and Dr Pat Upton (Veterinary College of Ireland) began to compute nutrient intakes for the Irish population using household budget surveys and retail price data. This led to a presentation on patterns of fatty acid intake in Ireland at the Nutrition Society in London for which he was complimented by Professor Robert McCance^(3)^. This unexpected support from such an eminent scientist buoyed him up for months, and the successful dabbling into population food intake data foreshadowed another road he would take in his later work.

Mike decided that his present post was a lost cause and started seeking an opportunity that could strengthen his credentials as an aspiring human nutrition researcher. He was initially offered a 2-year post in nutrition at the University of Southampton which appeared ideal except for the short-term nature of the contract. He wavered and decided it was too risky for the family, but Jo intervened knowing that his heart was in the Southampton post. In 1977, Mike joined the Medical School of Southampton University, where he would stay for 7 years enjoying the ‘majestic labs’ of Southampton and the company of academics and researchers from globally leading universities. He immersed himself in the world of mechanistic nutrition, including working with an animal model of human atherosclerosis which would give him his first paper in experimental biology (his ‘badge of honour’)^(4)^. But he also began to work with human volunteers investigating the effects of fibre on blood pressure, post-prandial lipids and measurement of human antibodies to dietary proteins. At Southampton, he became fully involved in the Nutrition Society, working with Bob Grimble as assistant programme secretary and immersing himself in the joys of regular science meetings around the UK.

Around this time, Mike started providing science articles to the *Guardian* newspaper, some of which included challenges to conventional wisdom on topics such as the role of antioxidants in CVD, which provoked a ‘snooty response from Sir Richard Doll’ but later proved to be a pretty accurate assessment. And so the scene was set for the full emergence of Mike Gibney – experimental scientist, energetic collaborator, scientific communicator and enthusiastic myth buster.

It was time to go home.

In 1984, Mike was offered and accepted a post at Trinity College, Dublin, in the Department of Clinical Medicine, where his work flourished, gaining the title of Professor of Nutrition and leading many successful collaborations, including his work on post-prandial lipid metabolism. Over the next few years, his persuasive charm, leadership and strong networks contributed to major developments in nutrition at the national level in Ireland. A meeting convened by Professor Gerry McKenna (University of Ulster) formalised a relationship between universities in Dublin, Ulster and Cork – the Irish Universities Nutrition Alliance, which has continued to be highly successful with strong research and teaching programmes funded nationally and via the European Union. The three leaders of the Irish Universities Nutrition Alliance, Mike Gibney, Sean Strain and Albert Flynn, worked together in great harmony forming not only a powerful scientific collaboration but a friendship that would last their lives. Mike called them the ‘Three Amigos’ who, to be sure, would be seen in great conviviality at most meetings of the Nutrition Society. The formation of the Irish Section of the Nutrition Society, which many had considered would take years to achieve, was established in 1988, propelled by the work of Dr Fred Andrews with Mike as co-plotter, and soon-to-be first chair of the Irish Section of the Nutrition Society. Mike’s membership in the Nutrition Society Council and his drive and leadership skills inevitably led to an invitation for him to become the next president of the Society. He readily accepted and served from 1995 to 1998 establishing working groups to carry out several strategic initiatives, which included developing stronger links with Europe, the formation of a Register of Nutritionists (now the Association for Nutrition) and the launch of a suite of nutrition textbooks aimed at undergraduate and post-graduate students. The latter he had described as ‘the craziest project I have ever undertaken’ proved to be very successful, generating income for the Society and a highly regarded set of textbooks now under the second editor-in-chief, Professor Sue Lanham-New.

At Trinity College, Mike launched himself into Europe, becoming a member of the European Scientific Committee on Food and of a collaboration known as Euronut led by Professor Jo Hautvast of the University of Wageningen, which aimed to encourage pan-European collaboration. Mike became a huge admirer of Jo Hautvast and vowed to emulate Jo’s style of leadership – positivity, vision and generosity. I was a beneficiary of Mike’s leadership with my own first big Framework grant in 1992, in which Mike was a collaborator. His support for me and enthusiasm for the work we were doing gave me confidence in making tough decisions and embedded my life-long respect and affection for this generous man. We collaborated again on the LIPGENE project, a much larger consortium with Mike as coordinator and Professor Julie Lovegrove as lead at the University of Reading. Mike’s wife Jo became the administrator on LIPGENE, proving to be an outstanding appointment as the go-to person for all the issues that arose during these complex and lengthy projects. Mike and Jo came as a pair, always succeeding in hitting deadlines, no matter how gruelling the barriers, bureaucracy and frustrations that work of this size can generate.

Mike’s move from Trinity College to University College Dublin in 2006, although controversial at the time, was part of his vision to advance the discipline of human nutrition – first, to exploit the development of metabolomics and data analytics to advance understanding of the effects of diet on human metabolism^(5)^ and, second, to bring nutrition into an academic environment where integration between relevant centres of excellence could allow solutions to big global challenges, such as climate change and obesity, to be developed. This bold and unexpected move to University College Dublin with his team to form the virtual Institute of Food and Health can be logically traced through his scientific career and his journey as a decisive leader, informed risk-taker and integrative nutrition scientist^(1)^.

Since ‘retiring’ Mike further developed his skills and inclination as a myth buster, offering a challenge to conventional thinking such as the benefits of a sugar tax on obesity^(6)^ and criticising the concept of ultraprocessed foods^(7)^. Mike did not direct his views to any particular vested interest – all have been the object of his active brain and incisive analyses. Wherever he saw sloppy thinking, lack of an evidence base, insufficient safety or impact data and, at times, the absence of sheer common sense, he would be inclined to ‘stick his head above the parapet’. He understood that some thought him wrong and excessively opinionated, but he remained an iconoclast to the last.

Mike collaborated with and provided advice to parts of the food industry. His repost to those who claim this could be a conflict of interest and a potential source of bias was brisk, ‘Scientific integrity is a necessary virtue of any researcher, and the only adjudicator of your compliance is your own conscience. Working with industry does not diminish science. Only you can do that, with whomsoever you collaborate’.

Mike’s ferocious work ethic, ambition and drive might suggest his attention to family matters could have been restricted. But he married Jo, and she made sure this could never be the case. They parented three children through the journey from Dublin to Sydney and back, then Southampton and back to Dublin, using the same love of conversation and conviviality that was evident in his working life. Their home was an open house to their friends and their children’s friends and to extended family, as well as to the many colleagues who became part of their lives. Eileen, Sinead and Michael are talented, successful, independent people with families of their own, whose love for their father shone through the extraordinary day of mourning and celebration of the life of their beloved father on 28 February 2024. The family were comforted in their loss by the hundreds of people who joined the congregation at St. John the Baptist’s Church, Blackrock, Dublin.

The three amigos have become two, and none of us will see his like again.
